# The Effect of ECAP Temperature on the Microstructure and Properties of a Rolled Rare Earth Magnesium Alloy

**DOI:** 10.3390/ma12091554

**Published:** 2019-05-12

**Authors:** Yun Tan, Wei Li, Weiwei Hu, Xiaofang Shi, Liang Tian

**Affiliations:** 1 College of Material and Metallurgy, Guizhou University, Guiyang 550025, China; tyl19941020@163.com (Y.T.); m18786670951@163.com (W.H.); 13017461042@163.com (X.S.); 2 Guizhou Province Technology Innovation Service Center, Guiyang 550004, China; 18285117265@163.com

**Keywords:** Mg-2Y-0.6Nd-0.6Zr alloy, equal channel angular pressing, predeformation, deformation temperature

## Abstract

Deformation of an as-rolled rare earth Mg-2Y-0.6Nd-0.6Zr alloy, at different temperatures, was carried out along the BC (90° anticlockwise rotation of the samples after each ECAP pass) route by equal channel angular pressing (ECAP). The effects of the deformation temperature and the predeformation on the microstructure of the magnesium alloy were determined by the microstructure examination. The slip systems and texture change of the Mg-2Y-0.6Nd-0.6Zr alloy were investigated by X-ray diffraction (XRD) and electron backscattered diffraction (EBSD), after equal channel angular deformation. The results showed that after seven passes of rolling, the grain size in the Mg-2Y-0.6Nd-0.6Zr alloy was refined to approximately 22 µm and the slip occurred mainly by a cylindrical slip and a pyramidal slip. After one pass of ECAP at 340 °C, the internal average grain size was significantly reduced to 11 µm, the cylindrical diffraction intensity clearly weakened, and the pyramidal diffraction intensity increased. EBSD pole figure analysis revealed that the base texture of the rolled Mg-2Y-0.6Nd-0.6Zr alloy weakened from 24.31 to 11.34 after ECAP. The mechanical properties indicated that the tensile strength and elongation of the rolled Mg-2Y-0.6Nd-0.6Zr alloy reached maximum values, when the deformation temperature was 340 °C.

## 1. Introduction

In recent years, to reduce energy consumption and environmental pollution caused by transportation, applications of new magnesium alloys, at high temperatures, have been studied by many researchers. Mg alloy is widely used in automobiles, aerospace, missiles, and other fields, because of its low density and high specific strength [1,2,3,4]. However, an inadequate number of slip planes at room temperature, results in poor workability, formability, and processing difficulty at room temperatures. Furthermore, the machining temperature of magnesium alloy is usually higher than 200 °C [5,6], which causes grain growth and a lower strength, due to the large grains. Therefore, it is important to improve the plasticity of magnesium alloys and their comprehensive properties [7,8,9,10].

Rare earth Mg alloys exhibit good strength and good creep resistance [11]. In particular, rare earth magnesium alloys show an improved high temperature performance, compared to conventional magnesium alloys, and are able to withstand service temperatures up to ~250 °C [12]. Avvari et al. [13] showed that the grain refinement of an AZ61 magnesium alloy decreased with increasing deformation temperature, but the elongation increased. Liang et al. [14] simulated the extrusion process of an AZ31 magnesium alloy. The results demonstrated that the temperature plays an important role in the extrusion process—it not only affects the structure evolution but also determines the surface quality of the extruded sample. Garces et al. [15] found that a pre-extrusion treatment can effectively refine grains, thereby, improving the plasticity of the alloy. Reducing the deformation temperature of the subsequent equal channel angular pressing (ECAP), not only enables ECAP to be performed at a lower temperature but also improves the plasticity. Previous studies regarding the magnesium alloys were mostly done for as-cast magnesium alloys, the plasticity and strength of the wrought magnesium alloys have been less studied. In particular, for rare earth wrought magnesium alloys, studies of the grain size, texture change, and material properties, after ECAP, are still lacking.

Herein, hot rolling was used as the pretreatment for the Mg-2Y-0.6Nd-0.6Zr alloy, and the effect of the deformation temperature and predeformation on the microstructural evolution and mechanical properties of the Mg-2Y-0.6Nd-0.6Zr alloy were investigated.

## 2. Materials and Methods

In this study, the raw materials comprised pure magnesium and Mg-25% Y, Mg-25% Nd, and Mg-30%Zr alloys. The pure magnesium components are listed in Table 1. The mass ratio was determined according to Mg-2Y-0.6Nd-0.6Zr. RJ-5 was composed of 56% anhydrous carnallite, 30% BaCl_2_, and 14% CaF_2_, which was used as a covering agent and a refining agent for melting. Before casting, the mold was preheated to 150 ± 10 °C, and a cylindrical metal rod of Φ 30 mm × 200 mm was cast and water-cooled, immediately after casting.

**Figure 1:**
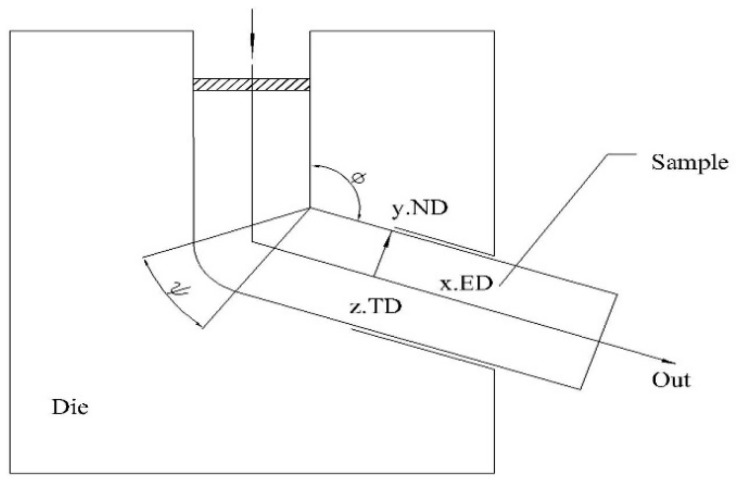
Equal channel angular pressing (ECAP) mold diagram.

The cast billet was homogenized at 450 °C for 6 h and then air-cooled. Cylindrical bars were hot rolled, 7 times, using an F50-150 rounding machine. The total strain from rolling was 0.46, and the alloy was preheated at 400 °C, for 15 min, before rolling. The bar was reheated to 400 °C and maintained at that temperature for 5–10 min. The ECAP deformation of the as-rolled magnesium alloy was performed at 300 °C, 320 °C, 340 °C, and 360 °C, using the ECAP mold structure shown in Figure 1. As shown, the die used for ECAP had two characteristic corner angles of φ = 120° and Ψ = 30°. The ECAP pressing was conducted via route BC (90° anticlockwise rotation of the samples after each ECAP pass) [16], and the specimens were lubricated with oil + graphite, inserted into the die, held until they reached the processing temperature, and then pressed at a constant rate of 0.4 mm/s.

The microstructure of the specimens was examined by optical microscopy (OM, Olympus, Japan). Samples for the OM investigation were ground and polished down with a 0.05 µm alumina slurry. The microstructure analyses were conducted on the planes perpendicular to the transverse direction (TD), after the samples were chemically etched with a solution of picric acid, nitric acid, ethanol, and water.

**Figure 2:**
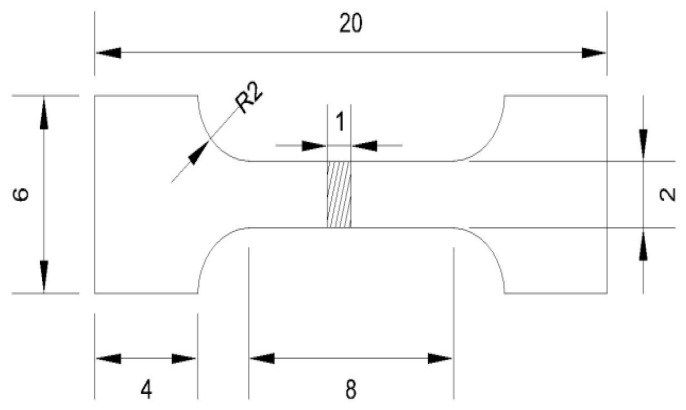
The dimension of the uniaxial tensile specimen (Unit: mm).

Uniaxial tensile tests at room temperature were performed using an Instron 8501 testing machine (Instron, Canton, USA). The tensile specimen were fabricated, according to the standards GB/T 228-2002, as shown in Figure 2. The texture was analyzed by X-ray diffraction (PANalytical B.V., Aermoluo, Holland) with CuKα radiation operated at 40 KV and 40 mA. The grain size was calculated from the micrographs, using Image-Pro Plus image analysis software. The tensile fracture of the sample was observed using a SUPRA 40 (ZEISS) scanning electron microscope (SEM, ZEISS, Oberkochen, Germany), with an acceleration voltage of 20 kV. Electron backscattered diffraction (EBSD) analysis was performed on a S-3400N scanning electron microscope (Hitachi, Tokyo, Japan), equipped with the TSL OIM Analysis 5 software package.

## 3. Results and Discussion

### 3.1. Effect of Predeformation Temperature on the Microstructure and Properties of the Alloy

**Figure 3:**
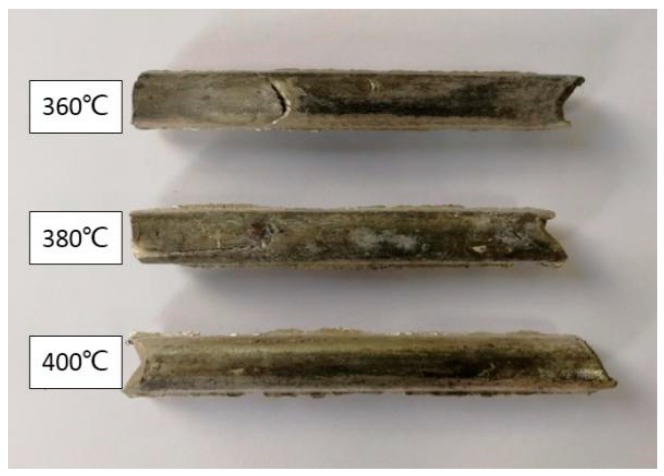
Photograph of rolled samples.

In the experiment, different rolling temperatures of 360 °C, 380 °C, and 400 °C were selected. A photograph of the samples after rolling is shown in Figure 3. Clearly, when the rolling temperature was 360 °C, cracks appeared on the surface of the sample, and the surface quality was poor. With an increasing rolling temperature, the surfaces of the samples were smoother, and the quality was better. Typically, it was difficult to initiate the slip systems in magnesium alloys at low temperatures, and the stress concentration caused shear fracture; if the temperature was too high, the grain size increased, and the plasticity affected the performance of the alloy.

**Figure 4:**
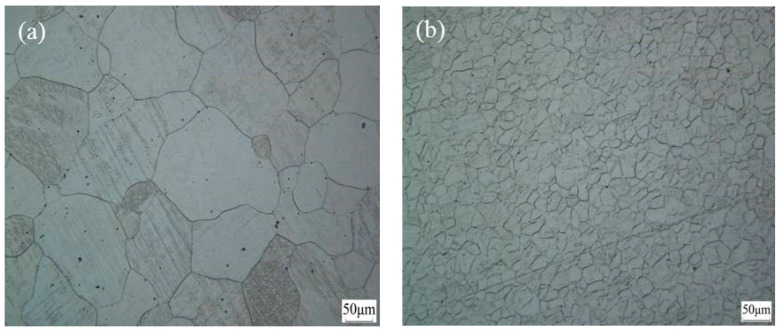
Optical microstructures of the Mg-2Y-0.6Nd-0.6Zr magnesium alloy: (**a**) As-cast state, and (**b**) as-rolled state.

Figure 4a presents the microstructure of the magnesium alloy in a uniform as-cast state. It appears that the sample was homogeneous and comprised large grains with a size of 85 μm. Moreover, the grain structure and grain boundaries were relatively clean and uniform. After seven rolling passes at 400 °C, the microstructure was finer than that in the as-cast state. As shown in Figure 4b, an average grain size of 22 μm was attained after rolling. Consequently, as the rolling temperature increased to 400 °C, the required activation energy for the dislocation slip was provided, and the critical resolved shear stress (CRSS) of the non-basal slip systems was reduced [17,18,19]. In addition, the non-basal slip systems were initiated, such that, the grains were mutually coordinated and deformed, during the rolling process, the macroscopic defects basically disappeared, and the appearance of the rolled samples was good.

Adding a small amount of rare earth elements to the magnesium alloys caused the stacking fault energy to improve and the alloy to become prone to cross-slip. During the deformation process, dislocation multiplication, and dislocation tangles occurred, and the density of dislocations and deformation energy storage increased, simultaneously. Non-basal slip, cross-slip, and dislocation climb occurred during deformation, which was favorable for a dynamic recrystallization; recrystallized grains grew around large grains, and most large grains were gradually replaced by the recrystallized fine grains. For the Mg-2Y-0.6Nd-0.6Zr alloy studied here, some of the second phase particles precipitated after high-temperature rolling, and the second phase particles were mostly distributed at the grain boundaries. It could be observed from Figure 4b that the grain refinement was substantial.

**Figure 5:**
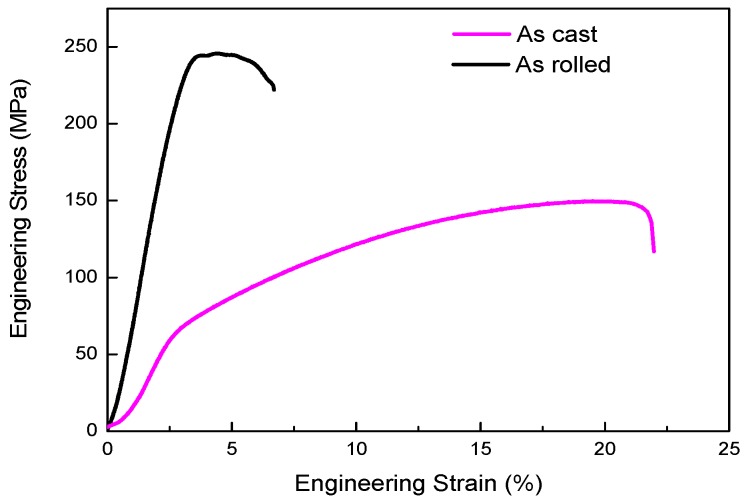
Mg-2Y-0.6Nd-0.6Zr alloy stress and strain curves of the as-cast and as-rolled states.

Figure 5 shows the true stress–strain curves of the samples, and the corresponding values of the ultimate tensile strength (UTS), 0.2% proof stress (YS), and tensile elongation to failure (TEF) are listed in Table 2. Compared with the as-cast sample, the seven-pass rolling procedure resulted in substantial increases in the UTS and YS, and a decrease in TEF, all of which were attributed to work hardening during rolling. The tensile strength improved by 61% from 150 MPa to 246 MPa, but the plasticity was significantly reduced, and the elongation was reduced by approximately 78%. Therefore, the as-rolled magnesium alloy exhibited a greater brittleness and higher strength than that of the as-cast alloy, but its plasticity was poor. This result occurred because the Mg-2Y-0.6Nd-0.6Zr alloy could not be recrystallized and softened, when rolling at 400 °C. As the deformation degree increased, the work hardening effect gradually increased, and grain refinement occurred. There were a large number of dislocations generated, so, when the rolling occurred, the YS of the alloy substantially improved and the TEF decreased.

### 3.2. Microstructure Evolution of Mg Alloy at Different Deformation Temperatures

**Figure 6:**
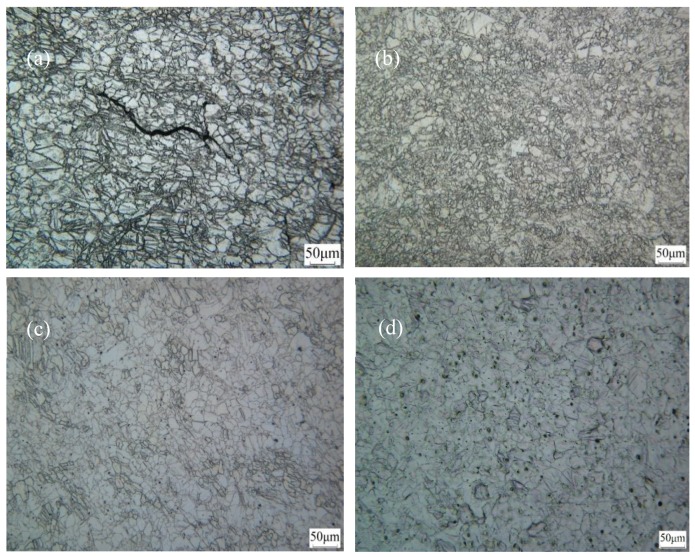
Optical microstructures of the deformed Mg-2Y-0.6Nd-0.6Zr alloy after ECAP at different temperatures: (**a**) 300 °C, (**b**) 320 °C, (**c**) 340 °C, and (**d**) 360 °C.

The microstructures of the samples after ECAP at different temperatures are shown in Figure 6. When the sample was extruded at a high temperature, a slight deformation occurred before entering the shearing zone, and then, severe plastic deformation occurred, after entering the shearing zone. The high temperature simultaneously provided nuclear driving forces for the equal-channel angular pressed wrought magnesium alloy. However, due to the increase in the dislocation density and increase in internal storage energy, dynamic recrystallization occurred in the shear band, and the crystal grains were refined [20,21,22]. When the deformation temperature of the ECAP process was 300 °C, a large number of twins appeared inside the coarse grains, and the structure was relatively uniform, as shown in Figure 6a. However, there were fine cracks in the core along the grain boundary. The potential slip systems of an as-rolled magnesium alloy were not usually activated at 300 °C, which made it difficult to initiate the deformation of the c-axis. When shear deformation along the (110) planes occurred as a result of the ECAP, it was difficult for each slip system to activate. Hence, there was a phenomenon of cracking along the crystal, which seriously affected its mechanical properties.

When ECAP at 320 °C was performed for 1 pass, as shown in Figure 6b, the recrystallized area increased, and the proportion of small grains increased, compared with that at 300 °C. However, the grain structure was inhomogeneous and consisted of elongated grains with relatively fine grains. At 340 °C, no twin structure was found, the alloy structure was further refined, the average grain size was approximately 11 µm, and the refining effect was better than that of the extrusion at 320 °C, for 1 pass, as shown in Figure 6c. As the temperature increased (as shown in Figure 6d), it was clear that the recrystallized grains began to grow and form a fully recrystallized microstructure.

### 3.3. Texture Evolution of the Mg Alloy at Different Temperatures

**Figure 7:**
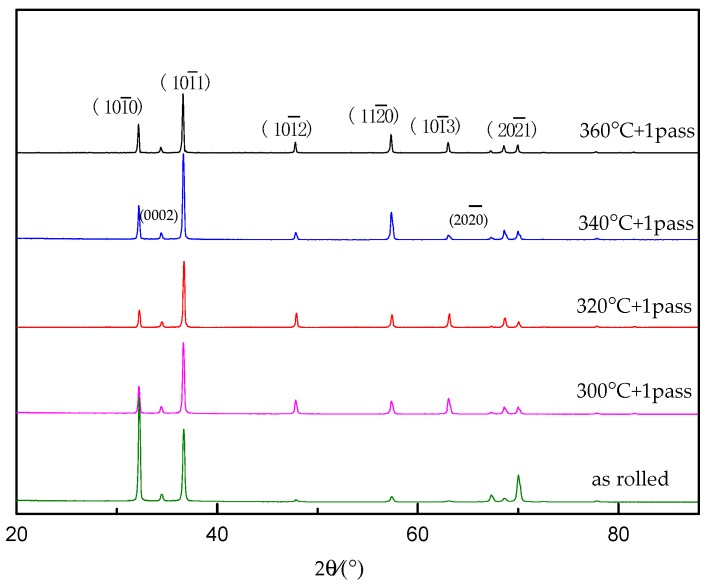
XRD spectra of Mg-2Y-0.6Nd-0.6Zr alloy during ECAP deformation at different temperatures.

Figure 7 presents the XRD pattern of the original sample and the extruded state after ECAP at different deformation temperatures. It could be observed from the figure that the strongest diffraction peak of the as-rolled sample was the {10$\bar{1}$0} peak, followed by the {10$\bar{1}$1} peak, which meant that the slip surface in the as-rolled sample had a clear preferred orientation, and a portion of the pyramidal slip system was activated at some point. However, after ECAP, the {10$\bar{1}$1} diffraction peak was significantly enhanced, and the {10$\bar{1}$0} diffraction peak was significantly weakened. In addition, the intensities of diffraction peaks, such as {10$\bar{1}$2}, {11$\bar{2}$0}, and {10$\bar{1}$3}, were enhanced after ECAP, which was due to more pyramidal slip systems being activated with the temperature increase. The main slipping mechanisms of the as-rolled sample were cylindrical pyramidal slips [23,24]. After ECAP, the intensity of the pyramidal slip system was clearly enhanced. This result occurred because the sample experienced a significant orientation change, after the ECAP deformation, which led to a decrease in the cylindrical orientation and an increase in the pyramidal orientation. Thus, the slip line helped the slip system to start, and the plasticity was also improved. At different ECAP deformation temperatures, the peak height of the Mg matrix phase in the sample, experienced almost no change, indicating that the original Mg-2Y-0.6Nd-0.6Zr alloy was slowly transformed from the typical rolled state texture to the equal-channel angular pressed texture, and the orientation did not change. The relationship between the strength of the material and the grain size, at this time, was in accordance with the Hall–Petch formula.

**Figure 8:**
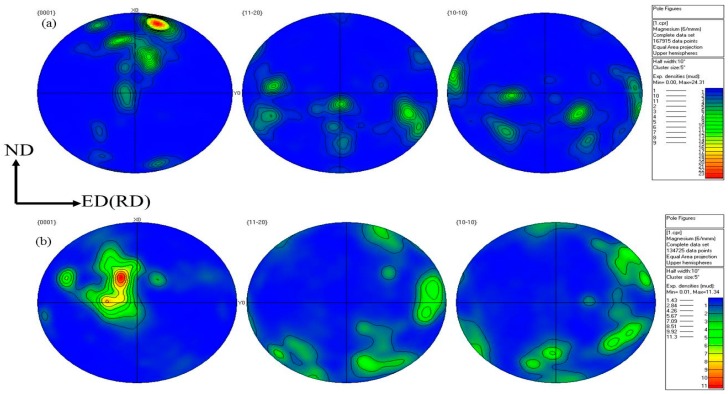
Electron backscattered diffraction (EBSD) analysis results: pole figures of the as-rolled state (**a**) and extruded state (**b**).

The pole figure of the as-rolled sample and the sample after ECAP at 340 °C, is shown in Figure 8. The as-rolled sample exhibited a strong basal texture of 24.31 and a texture along 15°, to the normal direction (ND), indicating that the direction of rolling and the basal (0001), occurred for a deflection of 15°. After a one-time extrusion at 340 °C, the basal texture intensity of the sample weakened from 24.31 to 11.34. As a result, due to the shear deformation introduced during the extrusion process, the orientation of the applied stress was changed, which could effectively change the texture of the magnesium alloy and promote texture weakening. This observation indicated that the basal texture could be appropriately reduced by ECAP.

**Figure 9:**
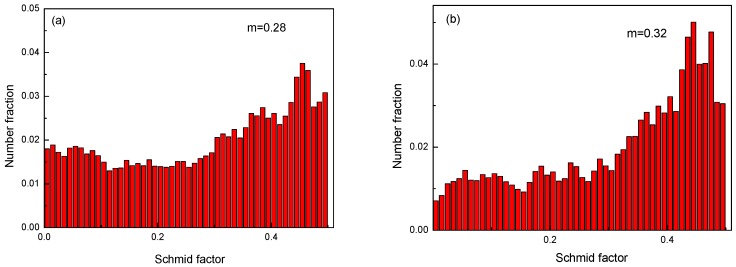
Schmid factor maps of the Mg-2Y-0.6Nd-0.6Zr alloy samples. (**a**) As rolled and (**b**) at 340 °C.

Consequently, to assess the effect of texture on strength, the Schmid factors were determined in terms of basal slip. Figure 9 shows the Schmid factor maps of the Mg-2Y-0.6Nd-0.6Zr alloy samples. The Schmid factor of the as-rolled sample was lower than that of the sample after ECAP, and the average Schmid factors were 0.28 and 0.32, respectively. Therefore, when the Schmid factor value was relatively high, more slip systems could be initiated in the alloy. The stress generated by the dislocation slip and the entanglement was dispersed, which decreased the tensile strength, as demonstrated for the sample after ECAP [25].

### 3.4. Mechanical Properties

**Figure 10:**
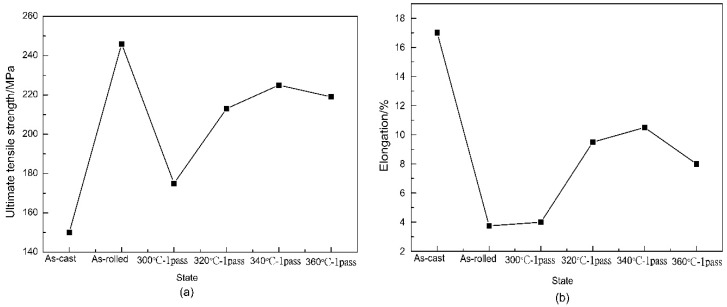
Tensile strength and elongation of the Mg-2Y-0.6Nd-0.6Zr alloy extruded by the ECAP, in a uniform state, at different temperatures: (**a**) Ultimate tensile strength, and (**b**) elongation.

It can be observed from Figure 10a that the UTS was improved, after one-pass extrusion at different temperatures, by ECAP. The corresponding values of the ultimate tensile strength (UTS) and tensile elongation to failure (TEF) are listed in Table 3. In particular, the UTS after ECAP was higher than that of the as-cast sample, but the TEF clearly decreased at 300 °C. This phenomenon might be due to the core cracks. Although the grain refinement was obvious, core cracking occurred, which substantially affected the tensile strength. When extruded at 340 °C for 1 pass, the mechanical properties improved, compared with that of the other pressing temperatures. The UTS and TEF at 340 °C reached the maximum values, and the UTS increased by 50%, compared with that of the as-cast samples. However, the UTS of the as-ECAPed samples decreased, relative to the as-rolled sample. According to the Hall–Petch relationship, the strength of the material should increase with a decreasing grain size, but its mechanical properties exhibited an anti-Hall–Petch relationship, after the ECAP deformation. Kim et al. [26] attributed this phenomenon to a texture softening effect greater than that of grain refinement, resulting in a decrease in the strength of the magnesium alloy. Jing et al. [27] also observed this phenomenon when studying the ECAP of the Mg-10.73Li-4.49Al-0.52Y alloy. Research has shown that the deformation of magnesium alloys by ECAP is affected by an increase of the grain boundaries, a new texture, and by grain refinement. Moreover, the effect of grain boundaries and texture softening was greater than that of grain refinement [28]. At the same time, it was also considered that during the ECAP process, the partial work hardening caused by rolling was eliminated.

**Figure 11:**
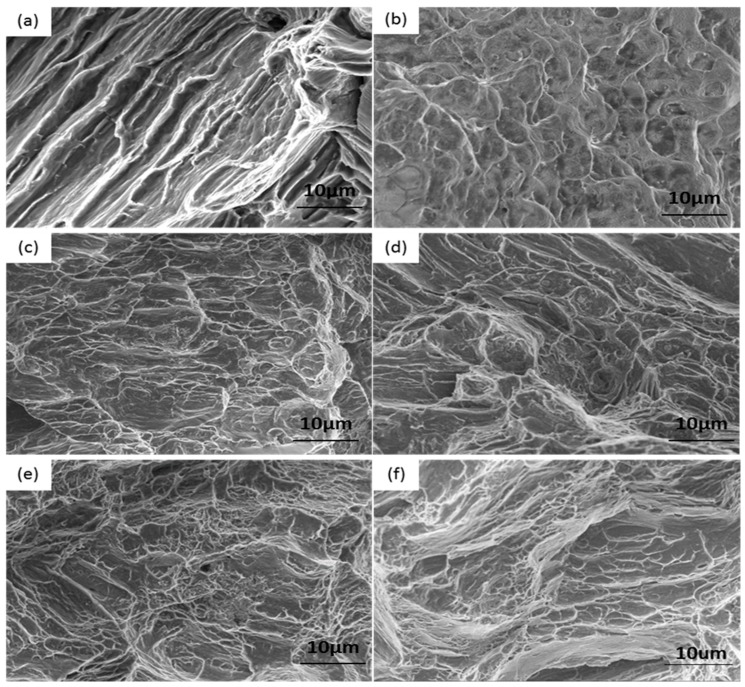
Tensile fracture SEM morphology of the ECAP extruded-rolled Mg-2Y-0.6Nd-0.6Zr alloy: (**a**) As cast, (**b**) as rolled, (**c**) 300 °C, (**d**) 320 °C, (**e**) 340 °C, and (**f**) 360 °C.

Figure 11 shows the fracture morphology of the Mg-2Y-0.6 Nd-0.6Zr magnesium alloy. It can be observed from Figure 11a that the as-rolled sample experienced brittle fracture and had shallow dimples, indicating a typical grain boundary fracture. After 1 pass of extrusion via ECAP, the area of a single grain boundary was reduced, and finer grains were formed. Moreover, the fracture mode changed from a brittle fracture to a ductile fracture, and a large number of dimples appeared on the tensile fracture surface. As shown in Figure 11d, the fracture surface exhibited a fluvial pattern, with a large dimple and a tearing ridge on the crystal surface, which indicated an intergranular toughness fracture.

## 4. Conclusions

1. Of the ECAP temperatures considered, the best rolling temperature of the Mg-2Y-0.6Nd-0.6Zr alloy was 400 °C. Compared with the as-cast sample, the average grain size of the rolled samples was refined from 85 µm to 22 µm, and the UTS improved from 150 MPa to 246 MPa, increasing by approximately 61%, compared with the as-cast magnesium alloy. However, the TEF was greatly reduced because of work hardening.
2. Compared with the as-cast sample, the sample after ECAP exhibited grain refinement with a minimum grain size of 11 µm, but the elongation increased, and the tensile strength substantially decreased. The decrease in tensile strength was due to the texture weakening and eliminating the effect of partial work hardening, after ECAP.
3. The sample processed with ECAP at 340 °C, achieved the best comprehensive properties, with a UTS of 225 MPa and a TEF of 10.5%. As a result, when the rare earth Mg-2Y-0.6Nd-0.6Zr alloy was subjected to ECAP, a suitable deformation temperature was 340 °C.

## Figures and Tables

**Table 1:** Chemical composition of pure magnesium (mass fraction/%).

Al	Zn	Mn	Ni	Fe	Cu	Mg
0.0061	0.0035	0.014	0.0004	0.0017	0.0035	Bal.

**Table 2:** The mechanical properties of the Mg-2Y-0.6Nd-0.6Zr alloy.

Sample	UTS (MPa)	YS (MPa)	TEF%
As-cast	150	64	17.00
As-rolled	246	216	3.75

**Table 3:** The mechanical properties of the samples.

State	UTS (MPa)	TEF%
As-cast	150.00	17.00
As-rolled	246.00	3.75
300 °C-1pass	175.00	4.00
320 °C-1pass	213.00	9.50
340 °C-1pass	225.00	10.50
360 °C-1pass	219.00	8.00
